# TIR1/AFB proteins: Active players in abiotic and biotic stress signaling

**DOI:** 10.3389/fpls.2022.1083409

**Published:** 2022-11-29

**Authors:** Wenchao Du, Yang Lu, Qiang Li, Shuangxia Luo, Shuxing Shen, Na Li, Xueping Chen

**Affiliations:** ^1^ Key Laboratory for Vegetable Germplasm Enhancement and Utilization of Hebei, Collaborative Innovation Center of Vegetable Industry in Hebei, College of Horticulture, Hebei Agricultural University, Baoding, China; ^2^ Hebei University Characteristic sericulture Application Technology Research and Development Center, Institute of Sericulture, Chengde Medical University, Chengde, China

**Keywords:** TIR1/AFB, abiotic stress, biotic stress, structural and functional specialization, transcription

## Abstract

The TIR1/AFB family of proteins is a group of functionally diverse auxin receptors that are only found in plants. TIR1/AFB family members are characterized by a conserved N-terminal F-box domain followed by 18 leucine-rich repeats. In the past few decades, extensive research has been conducted on the role of these proteins in regulating plant development, metabolism, and responses to abiotic and biotic stress. In this review, we focus on TIR1/AFB proteins that play crucial roles in plant responses to diverse abiotic and biotic stress. We highlight studies that have shed light on the mechanisms by which TIR1/AFB proteins are regulated at the transcriptional and post-transcriptional as well as the downstream in abiotic or biotic stress pathways regulated by the TIR1/AFB family.

## Introduction

Transport Inhibitor Response 1 and Auxin-Signaling F-box (TIR1/AFB) proteins are plant-specific receptors that mediate diverse responses to the plant hormone auxin (Dharmasiri et al., 2005; Parry et al., 2009). Upon binding indole-3-acetic acid (IAA), or other hormones in the auxin class, TIR1/AFB proteins form a co-receptor complex with Auxin/IAA (Aux/IAA) proteins (Salehin et al., 2015). Formation of this co-receptor complex results in ubiquitination and degradation of Aux/IAA proteins *via* the 26S proteasome (Pan et al., 2009; Salehin et al., 2015; Todd et al., 2020). Degradation of Aux/IAA proteins releases their inhibition of auxin response factors (ARFs), which are transcriptional regulators of auxin-responsive genes such as *Aux/IAA* (Strader and Zhao, 2016; Yu et al., 2022). In this way, TIR1/AFB proteins serve as positive regulators of downstream auxin-responsive pathways upon the perception of auxin (Quint and Gray, 2006; Dezfulian et al., 2016; Takato et al., 2017).

The first *TIR1/AFB* gene identified and shown to play an important role in auxin-regulated processes, such as hypocotyl elongation and lateral root formation, was *TIR1* in *Arabidopsis* (Ruegger et al., 1998). Subsequent studies identified *TIR1/AFB* family members encoded in the genomes of algae, mosses, and spermatophytes in addition to all land plants (Parry et al., 2009). The large number of *TIR1/AFB* genes encoded in plant genomes has allowed for functional redundancy and neofunctionalization to evolve (Prigge et al., 2020). It is now clear that TIR1/AFB proteins contribute to biological processes including regulation of primary and secondary metabolism (Gomes and Scortecci, 2021), seed and root development (Pan et al., 2009; Ozga et al., 2022), cell proliferation (Rast-Somssich et al., 2017), immunity and stress responses in plants (Iglesias et al., 2010). In this review, we highlight our current understanding of the structure and function of TIR1/AFB family members with an emphasis on possible mechanisms by which these proteins regulate abiotic and biotic stress responses.

## Structural and functional specialization of TIR1/AFB family members in *Arabidopsis*

Based on comparisons of land plant genomes sequenced to-date, TIR1/AFB proteins can be divided into four phylogenetic clades: TIR1/AFB1, AFB2/3, AFB4/5, and AFB6. *Arabidopsis* contains six TIR1/AFB proteins from three out of the four clades: TIR1, AFB1, AFB2, AFB3, AFB4, and AFB5 (Shimizu-Mitao and Kakimoto, 2014). AFB6 orthologs are noticeably absent in the core Brassicales species such as *Arabidopsis* as well as Poaceae species such as rice and maize (Prigge et al., 2020).

**Figure 1:**
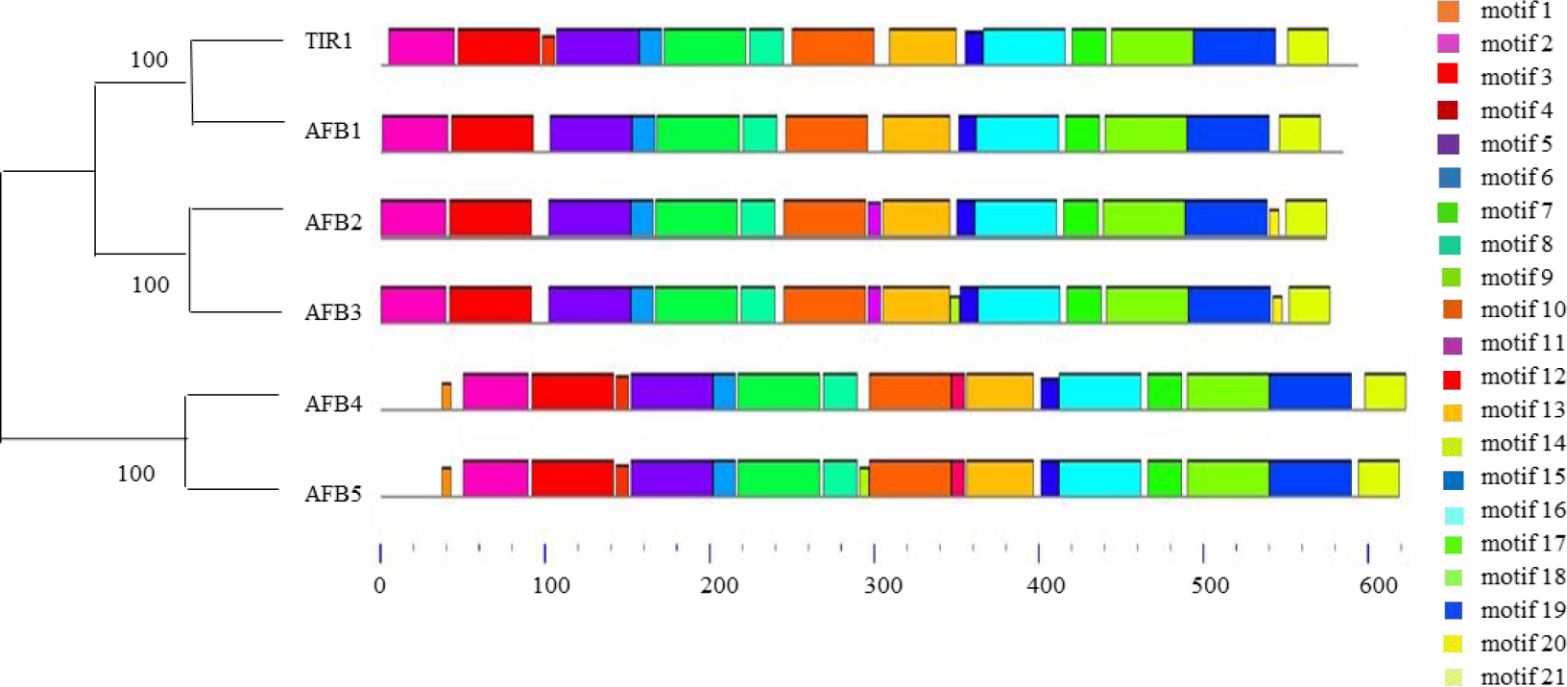
Neighbor-joining phylogenetic tree (left) and conserved motif (right) analysis of TIR1/AFBs in *Arabidopsis*.

The specific functions of TIR1/AFB family members vary considerably across and within clades. For instance, AFB4 and AFB5 are in the same clade yet exhibit distinct specificities for auxin (Prigge et al., 2016). Yeast two-hybrid and immunoblot assays demonstrated that IAA3 binds TIR1, AFB1, and AFB2 with different affinities but binds AFB5 very poorly at 0.1 μM IAA. Distinct motifs are necessary for the assembly of TIR1/AFB-IAA coreceptor complexes (Villalobos et al., 2012). Here, we generated a phylogenetic tree containing all TIR1/AFB family members from *Arabidopsis* and used Motif ENRichment Analysis (MEME) to identify conserved protein motifs (**Figure 1**). We believe the unique motifs present in TIR1/AFB proteins may explain their preferential binding of certain IAA proteins over others.

Our analysis showed that *Arabidopsis* TIR1/AFB proteins contain different conserved motifs. These proteins consist of a single F-box domain and eighteen LRR repeats (Prigge et al., 2020). F-box domains are critical for the regulated degradation of cellular proteins (Jain et al., 2007) while LRRs belong to an archaic procaryal protein architecture that is widely involved in protein-protein interactions (Martin et al., 2020). We found that different TIR1/AFB family members contain unique motifs. Motifs 1 and 12 are only present in AFB4 and AFB5, motifs 11 and 20 are only present in AFB2 and AFB3, motif 14 is only present in AFB3, and motif 9 is only found in AFB4. The presence and absence of certain motifs indicates that TIR1/AFBs may have different functions.

**Figure 2:**
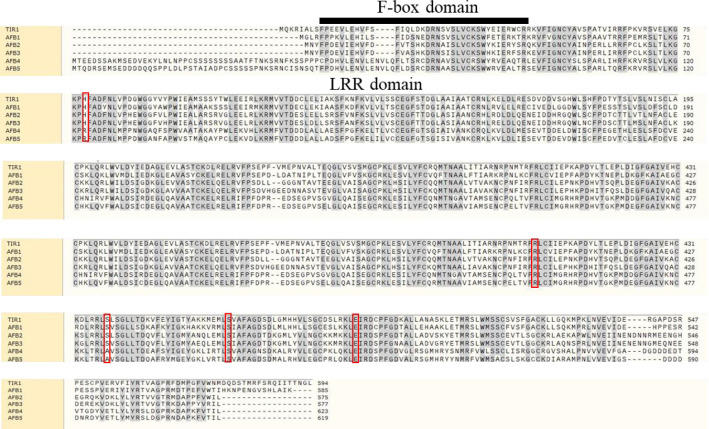
Multiple alignment of TIR1/AFB proteins in *Arabidopsis* adapted from Fu Guo et al. (Guo et al., 2021). Residues highlighted in gray are present in more than 50% of aligned sequences. The key residues making up the active site of the TIR1 pocket are highlighted by red boxes. Accession numbers of the genes encoding the proteins for the sequence alignment are as follows: TIR1 (*At3g62980*), AFB1 (*At4g03190*), AFB2 (*At3g26810*), AFB3 (*At1g12820*), AFB4 (*At4g24390*), and AFB5 (*At5g49980*).

Synthetic auxin herbicides are one of the most potent man-made abiotic stresses that plants are subjected to (Gorina et al., 2022). Picloram, 2,4-dichlorophenoxy acetic acid (2,4-D), and dicamba are three of the most widely used chemical classes of auxin. These herbicides function by binding to a hydrophobic pocket within TIR1/AFB proteins (Meng et al., 2008; Guo et al., 2021). Auxin binding TIR1 by filling in the bottom of TIR1 pocket, which floor is made up of several key residues containing His 78, Arg 403, Ser 438, Ser 462, and Glu 487 as shown in (**Figure 2**) (Guo et al., 2021). Distinct amino acid residues exist in the AFB4/5 clade compared with the TIR1/AFB1 and AFB2/3 clades at His 78 and Ser 438: histidine is replaced by arginine and serine is replaced by alanine. These differences demonstrate the diversity of TIR1/AFB members and suggest a structural reason for their specialized responses to different synthetic auxin herbicides.

Studies on *Arabidopsis* mutants have demonstrated that some members of the TIR1/AFB family are responsible for the recognition of specific auxin herbicides (Grossmann et al., 1996). For instance, the *Arabidopsis afb4/5* mutant is resistant to picloram whereas other *tir1/afb* mutants are still susceptible (Walsh et al., 2006). The AFB4 protein itself was shown to be a target of picloram based on *in vitro* binding assays (Prigge et al., 2016). TIR1 has been shown to be a receptor for 2,4-D and induces changes in gene expression when plants are treated with low concentrations of 2,4-D (Sheedy et al., 2006; Walsh et al., 2006). As anticipated, the *Arabidopsis tir1* mutant is resistant to 2,4-D whereas AFB1, a member of the same clade as TIR1, has not been implicated in 2,4-D resistance (Gleason et al., 2011).

*In vitro* assays demonstrated that TIR1 and AFB5 can bind to dicamba (de Figueiredo et al., 2022). Of all the *TIR1/AFB* family members in *Arabidopsis*, only the *tir1-1* and *afb5* mutants were shown to be resistant to dicamba (Gleason et al., 2011). No studies have yet implicated the AFB2/3 subgroup in auxin herbicide sensitivity, which further demonstrates the structural and functional specialization that exists in the TIR1/AFB family. However, studies on the rice mutants *Osabf2* and *Osabf3* showed *OsAFB2/3* genes are involved in the response to 2,4-D resistance (Guo et al., 2021). These results suggest that more studies should focus on the function of the AFB2/3 subgroup in herbicide susceptibility.

## The role of TIR1/AFB family members in abiotic and biotic stress responses

Plants are sessile organisms challenged by a variety of abiotic and biotic stresses from which they cannot escape. Abiotic stresses are caused by environmental conditions such as drought, high salinity, heat, and cold whereas biotic stresses are caused by living organisms such as bacteria, fungi, viruses, nematodes, and insects (Verma et al., 2016; Burns et al., 2018). Both abiotic and biotic stress induce reactive oxygen species (ROS) production in the form of hydroxyl radicals, hydrogen peroxide, and superoxide anions (Singh et al., 2020). At low concentrations, many ROS species function as signaling molecules in stress tolerance pathways. However, elevated and sustained levels of ROS can become toxic and lead to nutrient loss, resulting in metabolic disruption, abnormal hormone metabolism (Rejeb et al., 2014; Muchate et al., 2016), and growth inhibition (Gimenez et al., 2018). Auxin plays an indispensable role in how plants rapidly adapt to abiotic and biotic stress. As key auxin receptors in plants, the TIR1/AFB family has been shown to be essential for abiotic and biotic stress responses mediated by auxin.

### Drought stress

Drought is an important abiotic stress that negatively impacts plant development and results in reduced crop yield and quality. The expression of many *TIR1/AFB* genes is influenced by drought stress, which suggests the *TIR1/AFB* family may function in the drought tolerance pathway (Shu et al., 2015; Sharma et al., 2018; Benny et al., 2019). Over-expression and transcriptomic studies in *Populus trichocarpa*, *Arabidopsis thaliana*, *Oryza sativa*, *Zea mays*, *Solanum tuberosum*, *Triticum aestivum*, and *Agrostis stolonifera* have demonstrated that many *TIR1/AFB* genes are responsive to drought (Chen et al., 2012; Shu et al., 2015; Dalal et al., 2018; Sharma et al., 2018; Benny et al., 2019; Zhao et al., 2019). Relative water content (RWC) is used as a measure of plant water status and is a meaningful index of water stress tolerance (Lo Gullo and Salleo, 1988). *PtrFBL1* is a *TIR1* homolog in *Populus trichocarpa*. Overexpression of *PtrFBL1* in *P. trichocarpa* resulted in higher plant RWC values upon drought stress compared with non-transgenic plants (Shu et al., 2015).

Gene expression analyses suggest that some TIR1/AFB family members participate in drought responses in *Arabidopsis*. For example, TIR1 and AFB2 are required for the inhibition of lateral root growth by ABA or osmotic stress under drought stress (Chen et al., 2012). In seedling studies, *TIR1* was up-regulated under drought stress as determined by RNA-Seq (Benny et al., 2019). In addition to the well-studied *Arabidopsis* TIR1/AFB family, several TIR1/AFB proteins have also been implicated in drought responses in other species by transcriptional analysis. In rice, *TIR1* and *AFB2* expression levels were significantly downregulated in spikelets upon drought stress (Sharma et al., 2018). In maize and the Solanaceous crops tomato and potato, RNA-Seq results demonstrated that *TIR1* expression increased in seedlings exposed to drought stress (Benny et al., 2019). Drought-stressed roots of the wheat genotype viz. Raj3765 had increased expression of *AFB2*, suggesting *AFB2* may play a key role in response to drought (Dalal et al., 2018). Creeping bentgrass (*Agrostis stolonifera* L.) overexpressing the rice pri-miR393a exhibited improved tolerance to drought stress due to targeting and suppression of *AsAFB2* and *AsTIR1* expression (Zhao et al., 2019).

### Salt stress

Salt stress is a major environmental factor limiting plant growth and productivity. Salt stress can lead to ionic stress, osmotic stress, and secondary stresses such as oxidative stress (Yang and Guo, 2018). Mutant, overexpression, and ectopic expression studies of *TIR1/AFB* genes in *Arabidopsis* have uncovered a key role for some of these genes in salt stress tolerance. Expression of *AtNAC2*, which is typically induced by salt stress, is unresponsive to salt stress in the *tir1-1* mutant (He et al., 2005). An *Arabidopsis tir1afb2* double mutant exhibited enhanced tolerance against salt stress compared with wild-type plants as determined by a higher germination rate, greater root elongation, and higher chlorophyll content (Iglesias et al., 2010). The cucumber (*Cucumis sativus* L.) CsTIR1 and CsAFB1 proteins share 78% and 76% amino acid identity with their *Arabidopsis* homologs, respectively. However, ectopic overexpression of *CsTIR1* and *CsAFB1* in *Arabidopsis* led to higher germination and plant survival rates under salt stress (Chen et al., 2017). Over-expression of the *Arabidopsis AFB3* in *Arabidopsis* resulted in better primary and lateral root development and higher germination rates upon salt stress compared with the wild type (Garrido-Vargas et al., 2020).

It certainly seems contradictory that a *tir1afb2* double mutant and overexpression of *AFB3* or *CsTIR1/CsAFB1* both enhance salt stress resistance in *Arabidopsis*. This may be explained by increased activity of antioxidant enzymes in the *tir1afb2* mutant under salt stress. Higher levels of ABA are also detected in *tir1afb2* compared with wild-type plants (Iglesias et al., 2010) while more lateral roots are found in Arabidopsis transgenic lines overexpressing *AFB3*, *CsTIR1*, or *CsAFB1* (Chen et al., 2017; Garrido-Vargas et al., 2020). This may contribute to differential participation of TIR1/AFB family members and their tissue-specific functions (Iglesias et al., 2010; Garrido-Vargas et al., 2020).

In addition to numerous studies in *Arabidopsis*, TIR1/AFB proteins have also been implicated in salt stress responses in other plant species. Overexpression of maize *ZmAFB2* in tobacco led to enhanced salt tolerance (Yang et al., 2013). Eighteen *TIR1/AFB* genes have been identified in *Brassica juncea* var. tumida with qPCR analysis, which showed that some *BjuTIR1/AFB* genes are repressed by salt treatment (Cai et al., 2019). Degradome and miRNA sequencing analysis between salt-tolerant and salt-sensitive *Fraxinus velutina* Torr. tree cuttings demonstrated that reduced expression of *TIR1* by miR393a explains the enhanced salt stress tolerance of this tree species (Liu et al., 2022). Interestingly, AsAFB2 and AsTIR1 from creeping bentgrass may serve as a link between drought and salt stress response pathways, both pathways rely on ionic and osmotic homeostasis signaling (Zhu, 2002; Zhao et al., 2019), and AsAFB2 and AsTIR1 have been implicated involving in this process (Zhao et al., 2019). It is thus plausible that some TIR1/AFB family members may serve as key regulators of plant responses to multiple abiotic stresses.

### Temperature stress

Temperature is one of the most important environmental signals for plants. High and low temperatures have a variety of effects that affect plant growth and development profoundly (Sakamoto and Kimura, 2018). Expression data from different plant species indicates that members of the TIR1/AFB family participate in plant responses to temperature stress. For example, the *Arabidopsis tir1-1* mutant displays defective hypocotyl elongation at elevated temperatures (Gray et al., 2003). Expression of *TIR1/AFB2* in rice spikelets was significantly downregulated by heat stress, and the rice protein OsAFB6 can suppress flowering, which is thought to be a temperature sensor (He et al., 2018; Sharma et al., 2018). Finally, repression of *TIR1* expression in wheat impairs pollen exine formation in male sterility under cold stress (Liu et al., 2022).

### Phosphorus and nitrate stress

Phosphorus (Pi) and nitrate (a main source of inorganic nitrogen) are crucial nutrients for crop growth and development that are mainly absorbed from soil by roots. Phosphorous deficiency and excessive nitrate result in retardation of plant growth, development, and productivity (Koide et al., 1999; Zhang et al., 2017). The first TIR1/AFB protein found to be involved in Pi and nitrate availability is TIR1 from *Arabidopsis*, which was shown to be involved in pattern alterations of lateral root formation and emergence in response to phosphate availability (Perez-Torres et al., 2008). The expression level of *TIR1* is also induced under low Pi conditions (Mayzlish-Gat et al., 2012).

Regulation of root system architecture by external nitrate is mediated by AFB3 in *Arabidopsis* as demonstrated by *afb3* insertional mutants (Vidal et al., 2010). Integrated genomics, bioinformatics, and molecular genetics revealed that the expression of genes downstream of *AFB3* are influenced by external nitrate with the NAC4 transcription factor serving as a key regulator of this network (Vidal et al., 2013). AFB3-mediated activation of the two independent pathways in response to nitrate suggests that AFB3 is a unique nitrate response factor in *Arabidopsis* (Vidal et al., 2010). TIR1/AFB family members were also found to be key players in response to nitrate in other plant species. In *Lotus japonicus*, expression of *LjAFB6* is induced in response to exogenous nitrate (Rogato et al., 2021). These studies indicate that AFB3 in *Arabidopsis* and LjAFB6 in *L. japonicus* are potentially involved in plant responses to stress caused by excessive nitrate.

### Herbicide stress

Herbicides are small molecules that inhibit specific molecular target sites within plant biochemical pathways to affect physiological processes. Inhibition of these sites often has catastrophic consequences that are lethal to the plant (Dayan et al., 2010). Synthetic auxin, triazine, and organophosphorus herbicides are commonly used in agriculture to control weeds (Todd et al., 2020; Bigner et al., 2021; Striegel et al., 2021). Multiple members of the TIR1/AFB family are involved in susceptibility to synthetic auxin herbicides. Studies on *Arabidopsis TIR1/AFB* mutants have revealed a role for these genes in response to classical auxin herbicides. Recently, the *afb5* mutant was found to be resistant to a new auxin herbicide, halauxifen-methyl, which preferentially binds to AFB5 (Xu et al., 2022).

TIR1/AFB proteins also play a key role in the response to auxin herbicides in other plant species. In rice, CRISPR/Cas9 genome editing was used to generate *Ostir1/Osafb2/Osafb3/Osafb4/Osafb5* mutants that was resistant to 2,4-D. *Osafb4* mutants are highly resistant to the herbicide picloram (Guo et al., 2021). Expression of *TIR1* in wheat is clearly higher in *Triticum aestivum* than in *Aegilops tauschii*, resulting in less sensitive to the herbicide 2,4-D (Yu et al., 2021).

### Emerging evidence implicates TIR1/AFB proteins in metal stress tolerance and boron deficiency

In addition to the stresses described above, emerging evidence suggests that TIR1/AFB proteins may be involved in plant responses to metal, and boron deficiency. Aluminum toxicity inhibits plant growth and development (Liu et al., 2022). Inhibition of root morphogenesis under aluminum stress decreased in *Arabidopsis tir1* single and *tir1 afb2 afb3* triple mutants. Other genes in the auxin signaling pathway, such as ARFs, were also shown to be involved in aluminum sensitivity (Ruiz-Herrera and Lopez-Bucio, 2013). MicroRNAs targeting and mediating the cleavage of *TIR1/AFB* transcripts were shown to be essential for the aluminum stress response in *Arabidopsis* (Mendoza-Soto et al., 2012). These results suggest *TIR1*, *AFB2*, *AFB3*, and downstream auxin-responsive genes play an important role in aluminum sensitivity in *Arabidopsis*.

Boron is an abundant and essential micronutrient required by plants with deficiencies causing impaired plant growth (Park et al., 2005; Duran et al., 2018). Boron deficiency is positively correlated with the expression of many miRNAs. Gene expression analysis indicates that a subgroup of miRNAs regulate *TIR1/AFB* expression in Arabidopsis when boron is limited. This leads to decreased expression of *TIR1*, *AFB1*, and *AFB2* but increased expression of *AFB3* (Lu et al., 2015). Other reports have demonstrated that application of α-(phenylethyl-2-oxo)-indole-3-acetic acid (PEO-IAA), a synthetic antagonist of TIR1, could partially or fully restore cell elongation in boron deficient roots (Camacho-Cristobal et al., 2015).

## Biotic stress from pathogenic bacteria, fungi, viruses, nematodes, and phytophagous insects

Biotic stresses are mainly caused by pathogenic species of bacteria, fungi, viruses, nematodes, and insects that seek to acquire nutrients from their plant hosts (Jagdale and Joshi, 2019; Bhar et al., 2022). Damages caused by diseases and herbivory reduce crop yield and quality by affecting photosynthesis and secondary metabolite production in the host plant (Vo et al., 2021). Plants have evolved numerous strategies to defend themselves against these pathogens. These strategies rely on coordinated gene, protein, and hormone regulation to allow plants to sense and adapt to biotic stresses (Atkinson and Urwin, 2012). Auxin is a critical signaling component of the plant response to biotic stress, which suggests that TIR1/AFB proteins have a role to play as well (Ghanashyam and Jain, 2009; Bouzroud et al., 2018; Gidhi et al., 2022).

Plant pathogenic bacteria cause symptoms such as spots with yellow halos or mucus-like materials, which negatively impact agricultural production in many important crops (Zimaro et al., 2011). The tomato bacterial pathogen *Pseudomonas syringae* DC3000 (PtoDC3000) produces IAA to promote PtoDC3000 growth in plant tissues through suppression of SA-mediated host defenses (Wildermuth et al., 2001; McClerklin et al., 2018; Djami-Tchatchou et al., 2020). An *Arabidopsis tir1afb1 afb4 afb5* quadruple-mutant exhibited elevated IAA levels and reduced SA levels compared with WT (Djami-Tchatchou et al., 2020). An analysis of a *tir1* single mutant and *tir1 afb2 afb3* triple mutant revealed that these TIR1/AFB family members are targeted by diketopiperazines derived from *Pseudomonas aeruginosa* during colonization of *Arabidopsis* (Ortiz-Castro et al., 2011). The planar structure of diketopiperazines likely fits into the same pocket of TIR1 that synthetic auxins bind (Ortiz-Castro et al., 2011).

Fungal plant pathogens are ubiquitous, highly diverse, and can cause severe damage to many important crops (Termorshuizen, 2016). The *Arabidopsis afb1* and *afb3* mutants are partially resistant to the soilborne root pathogen *Verticillium dahlia*. Up-regulation of *pathogen-related gene 1* (*PR1*) in *afb1* and *pathogen defense factor 1.2* (*PDF1.2*) in *afb3* may be responsible for *afb1-* and *afb3*-mediated resistance, respectively (Fousia et al., 2018). Fusarium head blight (FHB) of wheat, caused by *Fusarium graminearum* Schwabe, results in large annual yield losses in wheat production regions. RNAi-mediated knockdown of the *TaTIR1* gene led to increased FHB resistance (Su et al., 2021). Gene expression studies also revealed that *TaTIR1* expression is highest at 24 and 48 h post-inoculation with the leaf rust pathogen *Puccinia triticina* Eriks (Gidhi et al., 2022). A maize *TIR1*-like gene is involved in the Zma-miR393b-mediated response to *Rhizoctonia solani* infection of leaf sheaths (Luo et al., 2014). Eighteen *TIR1/AFB* genes have been identified in *Brassica juncea* var. tumida using genome-wide analysis. qPCR analysis demonstrated that the expression of some *BjuTIR1/AFB* genes is influenced by *Plasmodiophora brassicae* infection (Cai et al., 2019).

Although no involvement in biotic stress has been reported for soybean TIR1/AFB proteins, TIR1/AFB proteins have been implicated in root nodulation induced by the nitrogen-fixing bacterium *Bradyrhizobium japonicum* (Cai et al., 2017). Overexpression of *GmTIR1* in soybean significantly increased the number of inflection foci and nodules while *GmAFB3A* may also play a minor role in this process (Cai et al., 2017).

Few studies to-date have implicated the TIR1/AFB family in plant defense responses against viruses. However, one study has shown that the rice dwarf virus (RDV) capsid protein P2 binds OsIAA10 and blocks the interaction between OsIAA10 and OsTIR1. This prevents 26S proteasome-mediated degradation of OsIAA10, resulting in plant dwarfism, increased tiller number, and short crown roots in infected plants (Jin et al., 2016).

Nematodes are pathogens of *Arabidopsis* (Moradi et al., 2021), apple (Fallahi et al., 1998), tomato (Khan and Khan, 1995), and wheat (Cortese et al., 2003), these species could move through roots and be vector of some virus, caused root damage, yield loss. The tomato *Mi-1* gene confers isolate-specific resistance against root-knot nematodes (Seah et al., 2007). Co-localization of TIR1-like proteins with the Mi-1 protein was observed (Seifi et al., 2011). *TIR1-*like transcript abundance in roots and leaves of nematode-resistant tomato lines was lower than in susceptible tomato lines, suggesting a possible role for *TIR1-*like genes in nematode resistance (Seifi et al., 2011).

Feeding by phytophagous insects such as aphids leads to reduced plant growth, reduced yield, water stress, dwarfism, wilting, and transmission of economically important plant viruses. In melon, genes like *TIR1* and *AFB2* are down-regulated in response to aphid herbivory. Application of the TIR1 inhibitor PEO-IAA to leaf discs resulted in significantly decreased feeding by aphids, providing *in vivo* support for TIR1/AFB in response to aphids (Sattar et al., 2016), suggested that TIR1 may play a role in aphid resistance.

## TIR1/AFB-regulated gene networks in abiotic and biotic stress responses

In addition to the regulation of *Aux/IAA* genes, many other proteins and genes regulated by TIR1/AFB family members have been identified that act downstream of auxin perception. These studies have contributed to our understanding of the mechanisms underlying the function of TIR1/AFB proteins in abiotic and biotic stress. These downstream genes and proteins include *nascent polypeptide-associated complex* (*NAC*) family members, SA synthesis proteins, PR proteins, PDF proteins and phosphorus transporters,

Auxin/indoleacetic acid (Aux/IAA) proteins play an important regulatory role in plant development and stress responses. TIR1/AFB proteins are essential regulators of the expression of a large number of *Aux/IAA* genes (Gray, 2003). For example, the rice Aux/IAA protein OsIAA20 mediates abiotic stress tolerance in rice through the ABA pathway (Zhang et al., 2021). Constitutive expression of *OsIAA18* in *Arabidopsis* led to improved salt and osmotic tolerance through enhanced ABA biosynthesis and ROS scavenging (Wang et al., 2021). The homeostatic expression of *Aux/IAA* is thought to be one of the most important resistance mechanisms to auxin herbicides mediated by TIR1/AFB proteins (Todd et al., 2020).

Aux/IAA proteins also play essential roles in response to biotic stress. Silencing of *GhIAA43* in cotton enhanced wilt resistance and activated the expression of SA-related defense genes (Su et al., 2022). Tobacco mosaic virus (TMV) replicase proteins negatively regulate IAA26 through a ubiquitin-mediated destabilization process to reduce TMV infection (Padmanabhan et al., 2005). The RDV capsid protein P2 can bind OsIAA10 directly, which implicates OsIAA10 in the defense response against RDV (Jin et al., 2016).

In addition to the *Aux/IAA* genes, many other stress-related genes are also regulated by TIR1/AFB proteins in response to abiotic and biotic stress. For example, the transcription factor NAC4 is an important positive regulator downstream of the AFB3 regulatory network, which plays an important role in the regulation of nitrate uptake in *Arabidopsis* (Vidal et al., 2013). The presence of a functional copy of *NAC1* is required by the fungal pathogen *Alternaria alternata* for full virulence in *Arabidopsis* (Wang et al., 2020). *NAC1* overexpression can restore lateral root formation in the *Arabidopsis tir1* mutant, whereas *TIR1* overexpression results in increased *NAC1* expression. These results demonstrate that NAC1 acts downstream of and can be positively regulated by TIR1 in *Arabidopsis* (Xie et al., 2000).

The SA-related genes *PR1* and *PDF1.2* are positive regulators of plant disease resistance that are negatively regulated by TIR1/AFB. A transcriptomic study in cotton demonstrated that knockdown of *GhTIR1* leads to a significant increase in the expression of SA-related genes in response to *Verticillium dahliae* infection (Shi et al., 2022). The *Arabidopsis* mutants *afb1* and *afb3* exhibit significantly higher expression of both *PR1* and *PDF1.2* in response to *Verticillium dahliae* infection (Fousia et al., 2018).

TIR1/AFB proteins act as mediators of low Pi uptake in *Arabidopsis* (Perez-Torres et al., 2008; Perez Torres et al., 2009). Pi deprivation increases the expression of *TIR1* in *Arabidopsis* seedlings (Perez-Torres et al., 2008). *ARF* was regulated by TIR1/AFB as described above. Knockout of *OsARF12* enhanced the expression of *PHOSPHATE TRANSPORTER1*(*PHT1*) genes such as *OsPHR2* in rice, suggesting that OsARF negatively regulates the *PHT1* gene family in rice (Wang et al., 2014).

## Regulation of TIR1/AFB expression and protein activity in response to abiotic and biotic stress

Many *TIR1/AFB* genes are differentially expressed in response to diverse abiotic or biotic stresses. Yet the underlying mechanism of *TIR1/AFB* gene regulation remains unknown.

*TIR1* expression is up-regulated or down-regulated in *Arabidopsis* upon infection by plant pathogens such as *Verticillium dahlia* and *Botrytis cinerea* (Llorente et al., 2008; Fousia et al., 2018). Many plant pathogens manipulate host auxin biosynthesis, inducing the degradation of AUX/IAA proteins through TIR1-mediated ubiquitination to enable greater infection (Wang et al., 2007). The *Arabidopsis* mutants *afb1* and *afb3* have enhanced plant resistance against *Verticillium dahlia.* However, the *tir1-1* mutant exhibits no increase in susceptibility to *Botrytis cinerea* compared to wild-type *Arabidopsis.* These studies indicate that TIR1/AFBs may be targeted by some pathogens.

Plant-produced small molecules are key systemic modulators of numerous biological pathways. Nitric oxide (NO) is an important signaling molecule involved in establishing resistance to plant stress. External NO represses *TIR1* expression and decreases *Arabidopsis* susceptibility to *Pseudomonas. syringae pv.* tomato: a process believed to be mediated by SA (Vitor et al., 2013). Hydrogen sulfide (H_2_S) is a gaseous molecule involved in various responses to stress. H_2_S negatively regulates the expression of *TIR1*, *AFB1*, *AFB2*, and *AFB3* in antibacterial resistance in *Arabidopsis* through a miR393a/b-regulated mechanism (Shi et al., 2015).

While most abiotic and biotic stresses suppress the expression of *TIR1/AFB* family members, some stresses can induce their expression. In *L. japonicus*, *LjAFB6* expression increased by 2.5-fold after nitrate treatment (Rogato et al., 2021). *Arabidopsis AFB3* was also found to be positively regulated by nitrate addition (Vidal et al., 2010; Vidal et al., 2013). Infections of *Plasmodiophora brassicae* in *Brassica juncea* var. tumida also induce the expression of *BjuTIR1/AFB* and *BjuTIR1* (Cai et al., 2019), but the mechanism by which this process occurs is not yet clear.

Some members of the *TIR1/AFB* family involved in abiotic or biotic stress responses are known targets of small RNAs. One of the most well-studied small RNAs shown to target and repress *TIR1/AFB* transcripts is MicroRNA393 (miR393) (Navarro et al., 2006). In *Arabidopsis*, miR393 directly targets *TIR1*, *AFB1*, *AFB2*, and *AFB2* transcripts in response to abiotic stress (Vidal et al., 2010; Chen et al., 2012; Iglesias et al., 2014). Regulation of *AFB3* by miR393 represents a unique nitrate-responsive module that is induced by nitrate and repressed by nitrogen metabolites in *Arabidopsis* (Vidal et al., 2010). Studies also indicate that miR393 negatively regulates *TIR1*, *AFB2*, and *AFB3* in response to pathogen challenge in several plant species (Navarro et al., 2006; Zhang et al., 2019; Shi et al., 2022). Though studies indicate that miR393 negatively regulates *TIR1* expression at the posttranscriptional level (Parry et al., 2009), the relationship between miR393 and *TIR1/AFB* transcripts needs to be investigated further.

In addition to regulated gene expression or posttranscriptional level, TIR1/AFB proteins are also regulated post-translationally by other proteins. The *Arabidopsis* TIR1 protein is stabilized by a complex consisting of heat shock protein 90 (HSP90) and Suppressor of G2 allele of skp1 (SGT1b), which itself is an HSP90 co‐chaperone, co-immunoprecipitation analyses further validated that HSP90 interacted with TIR1 (Watanabe et al., 2016; Munoz et al., 2022). So far, no other factors were found to positively or negatively regulate TIR1/AFB proteins at post-translational level under stress. Therefore, future study should explore factors that regulate or interact with TIR1/AFB proteins.

## Conclusions and perspectives

**Figure 3:**
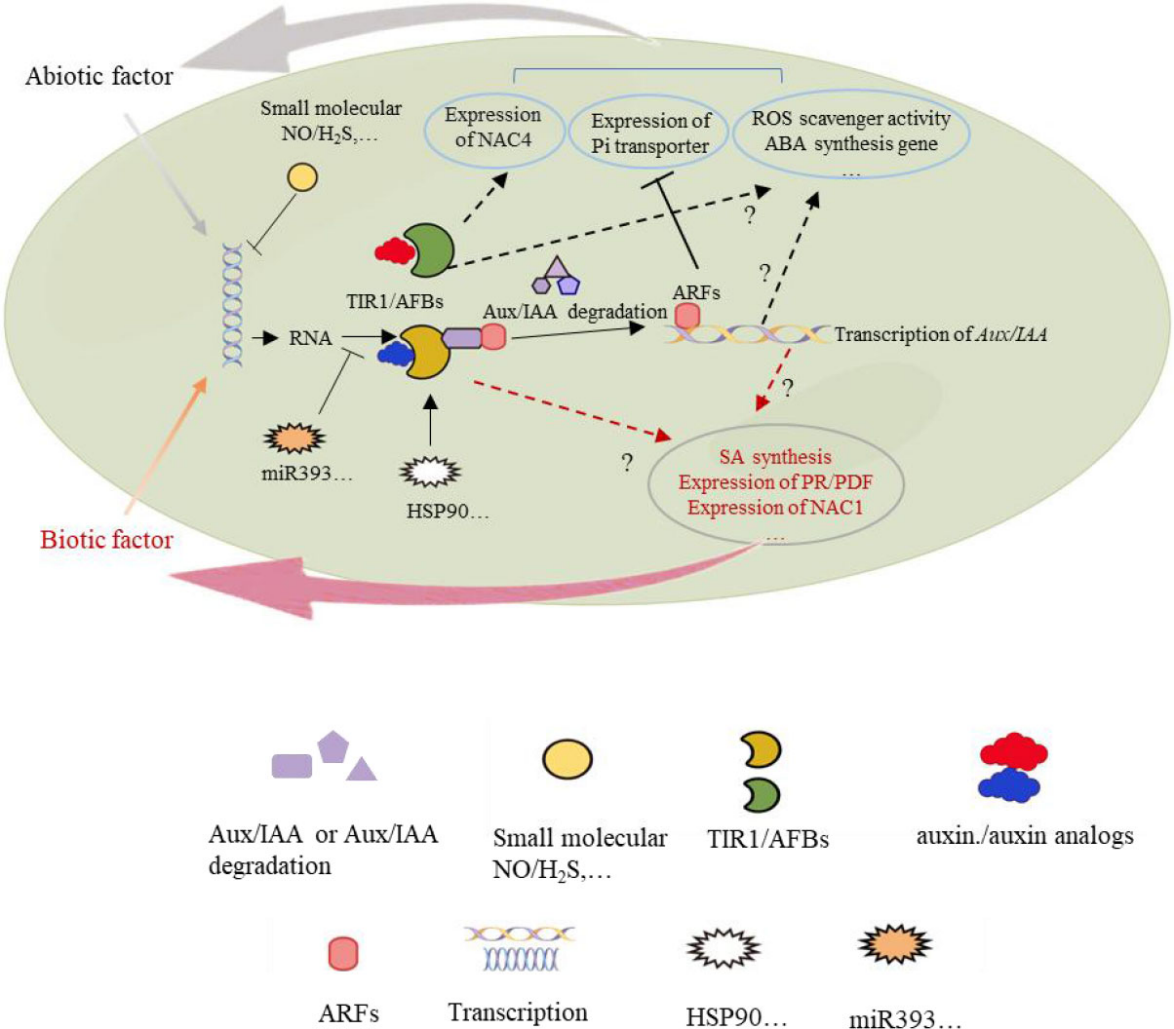
TIR1/AFB regulatory network in response to abiotic and biotic stress. TIR1/AFBs response to abiotic or biotic factors in different signal transduction pathways.

Phylogenetic, structural, and functional studies have revealed that there are many homologs of TIR1/AFB proteins with conserved domains. Many *TIR1/AFB* genes are differentially expressed in response to diverse abiotic and biotic stress (**Table 1**). Small molecules such as NO and H_2_S regulate *TIR1/AFB* gene expression, MicroRNAs, such as miR393, are some of the most well-studied regulators of *TIR1/AFB* transcripts. The regulation of some TIR1/AFB family members through protein-protein interactions and small molecules is also indispensable (**Figure 3**). Future studies should focus on identifying more factors that can regulate TIR1/AFB family members at the transcriptional, post-transcriptional, and protein levels. These studies will shed light on the evolution of the TIR1/AFB family and identify new roles for these proteins in plant abiotic and biotic stress responses.

**Table 1:** TIR1/AFB proteins involved in abiotic and biotic stress in plants.

Plant species	Name	Subfamily	stress	reference
Arabidopsis (*Arabidopsis thaliana*)	AtTIR1	TIR1/AFB1	salt	(Chen et al., 2015)
	AtAFB2	AFB2/3	Salt	(Iglesias et al., 2010)
	AtAFB3	AFB2/3	Salt	(Iglesias et al., 2010)
	AtTIR1	TIR1/AFB1	Temperature	(Wang et al., 2016)
	AtTIR1	TIR1/AFB1	Drought	(Chen et al., 2012)
	AtAFB2	AFB2/3	Drought	(Benny et al., 2019)
	AtTIR1	TIR1/AFB1	Low Pi	(Perez-Torres et al., 2008; Mayzlish-Gat et al., 2012)
	AtAFB3	AFB2/3	Nitrate	(Vidal et al., 2010; Vidal et al., 2013)
	AtTIR1	TIR1/AFB1	Herbicide	(Sheedy et al., 2006; Walsh et al., 2006; Gleason et al., 2011)
	AtAFB4	AFB4/5	Herbicide	(Gleason et al., 2011)
	AtAFB5	AFB4/5	Herbicide	(Gleason et al., 2011; Xu et al., 2022)
	AtTIR1	TIR1/AFB1	Aluminum	(Ruiz-Herrera and Lopez-Bucio, 2013)
	AtAFB2	AFB2/3	Aluminum	(Ruiz-Herrera and Lopez-Bucio, 2013)
	AtAFB3	AFB2/3	Aluminum	(Ruiz-Herrera and Lopez-Bucio, 2013)
	At TIR1	TIR1/AFB1	Boron deficiency	(Camacho-Cristobal et al., 2015; Lu et al., 2015)
	AtTIR1/AFB1/AFB4/AFB5	TIR1/AFB1, AFB4/5	bacterium	(Djami-Tchatchou et al., 2020)
	AtAFB1	TIR1/AFB1	Fungi	(Fousia et al., 2018)
	AtAFB3	AFB2/3	Fungi	(Fousia et al., 2018)
	AtTIR1	TIR1/AFB1	Fungi	(Ortiz-Castro et al., 2011)
	AtTIR1/AFB2/AFB3	TIR1/AFB	Fungi	(Ortiz-Castro et al., 2011)
Rice (*Oryza sativa*)	OsTIR1	TIR1/AFB1	Salt	(Xia et al., 2012)
	OsAFB2	AFB2/3	Salt	(Xia et al., 2012)
	OsAFB2	AFB2/3	Drought	(Xia et al., 2012; Sharma et al., 2018)
	OsTIR1	TIR1/AFB1	Drought	(Xia et al., 2012; Sharma et al., 2018)
	OsTIR1	TIR1/AFB1	Temperature	(Sharma et al., 2018)
	OsAFB2	AFB2/3	Temperature	(Sharma et al., 2018)
	OsAFB6	AFB6	Temperature	(He et al., 2018)
	OsTIR1	TIR1/AFB1	Herbicide	(Guo et al., 2021)
	OsAFB2	AFB2/3	Herbicide	(Guo et al., 2021)
	OsAFB3	AFB2/3	Herbicide	(Guo et al., 2021)
	OsAFB4	AFB4/5	Herbicide	(Guo et al., 2021)
	OsAFB5	AFB4/5	Herbicide	(Guo et al., 2021)
	OsTIR1	TIR1/AFB1	Virus	(Jin et al., 2016)
Wheat (*Triticum aestivum*)	TaAFB2	AFB2/3	Drought	(Dalal et al., 2018)
	TaTIR1	TIR1/AFB1	Temperature	(Liu et al., 2022)
	TaTIR1	TIR1/AFB1	Herbicide	(Yu et al., 2021)
	TaTIR1	TIR1/AFB1	Fungi	(Su et al., 2021)
	TaTIR1	TIR1/AFB1	Fungi	(Gidhi et al., 2022)
Maize (*Zea mays*)	ZmAFB2	AFB2/3	Salt	(Yang et al., 2013)
	ZmTIR1	TIR1/AFB1	Drought	(Benny et al., 2019)
	ZmTIR-like	TIR1/AFB1	Fungi	(Luo et al., 2014)
Soybean (*Glycine max L.*)	GmTIR1	TIR1/AFB1	Fungi	(Cai et al., 2017)
	GmAFB3	AFB2/3	Fungi	(Cai et al., 2017)
Melon (*Cucumis melo L*.)	CmTIR1	TIR1/AFB1	Aphid	(Sattar et al., 2016)
	CmAFB2	AFB2/3	Aphid	(Sattar et al., 2016)
Cucumber (*Cucumis sativus L.)*	CSTIR1	TIR1/AFB1	Salt	(Chen et al., 2017)
	CsAFB2	AFB2/3	Salt	(Chen et al., 2017)
Tomato (*Solanum lycopersicum*)	SlTIR1	TIR1/AFB1	Drought	(Benny et al., 2019)
	SlTIR1	TIR1/AFB1	Nematode	(Seah et al., 2007; Seifi et al., 2011)
Potato (*Solanum tuberosum*)	StTIR1	TIR1/AFB1	Drought	(Benny et al., 2019)
Mustard (*Brassica juncea* var. *tumida)*	BjuTIR1	TIR1/AFB1	Salt	(Cai et al., 2019)
	BjuAFB3		Salt	(Cai et al., 2019)
	BjuTIR1	TIR1/AFB1	Fungi	(Cai et al., 2019)
Crowtoe (*Lotus corniculatus* L.*)*	LjAFB6	AFB6	nitrate	(Rogato et al., 2021)
Creeping bentgrass (*Agrostis stolonifera L.)*	AsTIR1	TIR1/AFB1	Salt	(Zhao et al., 2019)
	AsAFB2	AFB2/3	Salt	(Zhao et al., 2019)
Fraxinus tomentosa (*Fraxinus velutina* Torr.)	FvTIR1	TIR1/AFB1	Salt	(Liu et al., 2022)

TIR1/AFB proteins are known regulators of numerous stress-related genes. The most well-studied examples of gene regulation by TIR1/AFB proteins are the *Aux/IAA* genes. Expression of many *Aux/IAA* genes in response to abiotic and biotic stress is both directly and indirectly controlled by TIR1/AFB proteins. Expression of *NAC4* is also regulated by TIR1/AFB proteins in response to nitrate uptake.

The general mechanism by which TIR1/AFB proteins enhance abiotic stress tolerance is by reducing ABA accumulation, increasing the abundance of ROS scavengers, and affecting the activity of other factors such as Pi transporters. In response to biotic stress, TIR1/AFB proteins promote the expression of SA biosynthesis genes, *PR* genes, and *PDF* genes. However, more studies need to be performed to determine the role of specific TIR1/AFB members in the signaling and metabolic pathways that modulate disease resistance. As the studies highlighted in this review demonstrate, much knowledge about the role of TIR1/AFB proteins in abiotic and biotic stress responses has been generated. The next challenge for the field will be deciphering the upstream and downstream events to draw a more complete picture of TIR1/AFB-mediated regulation of plant abiotic and biotic stress responses.

## Author contributions

XC and NL concepted the topic of this manuscript and revised the manuscript, WD drafted this manuscript with YL. QL, SL and SS revised the manuscript. All authors contributed to the article and approved the submitted version.

## Funding

This work was supported by the National Natural Science Foundation of China (grant no. 32172567), Vegetable Innovation Team Project of Hebei Modern Agricultural Industrial Technology System (grant no. HBCT2018030203), Key Research & Development Project of Hebei Province (grant no. 21326309D), The Innovation Ability Training Project for Graduate Student of Hebei Province (grant no. CXZZBS2018114), and the grant from ‘Giant Plan’ of Hebei Province.

## Acknowledgments

We thank A&L Scientific Editing (www.alpublish.com) for its linguistic assistance during the preparation of this manuscript. We also thank Ma Wei, Lisong Ma, Lijun Song and Shiyao You in the preparation of the pictures in this manuscript.

## Conflict of interest

The authors declare that the research was conducted in the absence of any commercial or financial relationships that could be construed as a potential conflict of interest.

## Publisher’s note

All claims expressed in this article are solely those of the authors and do not necessarily represent those of their affiliated organizations, or those of the publisher, the editors and the reviewers. Any product that may be evaluated in this article, or claim that may be made by its manufacturer, is not guaranteed or endorsed by the publisher.

## References

[B1] Atkinson N. J. Urwin P. E. (2012). The interaction of plant biotic and abiotic stresses: From genes to the field. J. Exp. Bot. 63, 3523–3543. doi: 10.1093/jxb/ers100 22467407

[B2] Benny J. Pisciotta A. Caruso T. Martinelli F. (2019). Identification of key genes and its chromosome regions linked to drought responses in leaves across different crops through meta-analysis of RNA-seq data. BMC Plant Biol. 19, 1–18. doi: 10.1186/s12870-019-1794-y 31077147 PMC6511156

[B3] Bhar A. Chakraborty A. Roy A. (2022). Plant responses to biotic stress: Old memories matter. Plants-Basel 11, 84. doi: 10.3390/plants11010084 PMC8747260 35009087

[B4] Bigner J. A. Fiester S. E. Fulcher J. W. Schammel C. M. G. Ward M. E. Burney H. E. . (2021). Glyphosate and polyoxyethyleneamine ingestion leading to renal, hepatic, and pulmonary failure. Am. J. Foren. Med. Path. 42, 282–285. doi: 10.1097/PAF.0000000000000660 33491949

[B5] Bouzroud S. Gouiaa S. Hu N. Bernadac A. Mila I. Bendaou N. . (2018). Auxin response factors (ARFs) are potential mediators of auxin action in tomato response to biotic and abiotic stress (*Solanum lycopersicum*). PloS One 13, 1–20. doi: 10.1371/journal.pone.0193517 PMC5831009 29489914

[B6] Burns E. E. Keith B. K. Refai M. Y. Bothner B. Dyer W. E. (2018). Constitutive redox and phosphoproteome changes in multiple herbicide resistant *Avena fatua* l. are similar to those of systemic acquired resistance and systemic acquired acclimation. J. Plant Physiol. 220, 105–114. doi: 10.1016/j.jplph.2017.11.004 29169105

[B7] Cai Z. M. Wang Y. N. Zhu L. Tian Y. P. Chen L. Sun Z. X. . (2017). GmTIR1/GmAFB3-based auxin perception regulated by miR393 modulates soybean nodulation. New Phytol. 215, 672–686. doi: 10.1111/nph.14632 28598036

[B8] Cai Z. M. Zeng D. E. Liao J. J. Cheng C. H. Sahito Z. A. Xiang M. Q. . (2019). Genome-wide analysis of auxin receptor family genes in *brassica juncea* var. tumida. Genes 10, 165. doi: 10.3390/genes10020165 30791673 PMC6410323

[B9] Camacho-Cristobal J. J. Martin-Rejano E. M. Herrera-Rodriguez M. B. Navarro-Gochicoa M. T. Rexach J. Gonzalez-Fontes A. (2015). Boron deficiency inhibits root cell elongation *via* an ethylene/auxin/ROS-dependent pathway in arabidopsis seedlings. J. Exp. Bot. 66, 3831–3840. doi: 10.1093/jxb/erv186 25922480 PMC4473985

[B10] Chen Z. Hu L. Han N. Hu J. Yang Y. Xiang T. . (2015). Overexpression of a miR393-resistant form of transport inhibitor response protein 1 (mTIR1) enhances salt tolerance by increased osmoregulation and na^+^ exclusion in arabidopsis thaliana. Plant Cell Physiol. 56, 73–83. doi: 10.1093/pcp/pcu149 25336111

[B11] Chen H. Li Z. Xiong L. (2012). A plant microRNA regulates the adaptation of roots to drought stress. FEBS Lett. 586, 1742–1747. doi: 10.1016/j.febslet.2012.05.013 22613571

[B12] Chen Z. H. Li M. T. Yuan Y. Hu J. Q. Yang Y. J. Pang J. L. . (2017). Ectopic expression of cucumber (*Cucumis sativus* l.) CsTIR/AFB genes enhance salt tolerance in transgenic arabidopsis. Plant Cell Tiss. Org. 131, 107–118. doi: 10.1007/s11240-017-1267-7

[B13] Cortese M. R. Fanelli E. De Giorgi C. (2003). Characterization of nematode resistance gene analogs in tetraploid wheat. Plant Sci. 164, 71–75. doi: 10.1016/S0168-9452(02)00336-9

[B14] Dalal M. Sahu S. Tiwari S. Rao A. R. Gaikwad K. (2018). Transcriptome analysis reveals interplay between hormones, ROS metabolism and cell wall biosynthesis for drought-induced root growth in wheat. Plant Physiol. Bioch. 130, 482–492. doi: 10.1016/j.plaphy.2018.07.035 30081325

[B15] Dayan F. E. Duke S. O. Grossmann K. (2010). Herbicides as probes in plant biology. Weed Sci. 58, 340–350. doi: 10.1614/WS-09-092.1

[B16] de Figueiredo M. R. A. Kupper A. Malone J. M. Petrovic T. de Figueiredo, A.B.T.B. Campagnola G. . (2022). An in-frame deletion mutation in the degron tail of auxin coreceptor IAA2 confers resistance to the herbicide 2,4-d in sisymbrium orientale. PNAS 119, e2105819119. doi: 10.1073/pnas.2105819119 35217601 PMC8892348

[B17] Dezfulian M. H. Jalili E. Roberto D. K. A. Moss B. L. Khoo K. Nemhauser J. L. . (2016). Oligomerization of SCF^TIR1^ is essential for Aux/IAA degradation and auxin signaling in arabidopsis. PloS Genet. 12, e1006301. doi: 10.1371/journal.pgen.1006301 27618443 PMC5019376

[B18] Dharmasiri N. Dharmasiri S. Estelle M. (2005). The f-box protein TIR1 is an auxin receptor. Nature 435, 441–445. doi: 10.1038/nature03543 15917797

[B19] Djami-Tchatchou A. T. Harrison G. A. Harper C. P. Wang R. H. Prigge M. J. Estelle M. . (2020). Dual role of auxin in regulating plant defense and bacterial virulence gene expression during *Pseudomonas syringae* PtoDC3000 pathogenesis. Mol. Plant Microbe In. 33, 1059–1071. doi: 10.1094/MPMI-02-20-0047-R PMC7810136 32407150

[B20] Duran C. Arce-Johnson P. Aquea F. (2018). Methylboronic acid fertilization alleviates boron deficiency symptoms in arabidopsis thaliana. Planta 248, 221–229. doi: 10.1007/s00425-018-2903-0 29700610

[B21] Fallahi E. Hafez S. L. Colt W. M. Seyedbagheri M. M. (1998). Effects of metam sodium and rootstock on plant-parasitic nematodes, tree growth, meld, fruit quality, and leaf minerals in 'braeburn' apple. Nematropica 28, 71–79.

[B22] Fousia S. Tsafouros A. Roussos P. A. Tjamos S. E. (2018). Increased resistance to *Verticillium dahliae* in arabidopsis plants defective in auxin signalling. Plant Pathol. 67, 1749–1757. doi: 10.1111/ppa.12881

[B23] Garrido-Vargas F. Godoy T. Tejos R. O'Brien J. A. (2020). Overexpression of the auxin receptor AFB3 in arabidopsis results in salt stress resistance and the modulation of NAC4 and SZF1. Int. J. Mol. Sci. 21, 9528. doi: 10.3390/ijms21249528 33333760 PMC7765236

[B24] Ghanashyam C. Jain M. (2009). Role of auxin-responsive genes in biotic stress responses. Plant Signal. Behav. 4, 846–848. doi: 10.4161/psb.4.9.9376 19847104 PMC2802801

[B25] Gidhi A. Mohapatra A. Fatima M. Jha S. K. Kumar M. Mukhopadhyay K. (2022). Insights of auxin signaling f-box genes in wheat (Triticum aestivum l.) and their dynamic expression during the leaf rust infection. Protoplasma. 1–17. doi: 10.1007/s00709-022-01808-4 36100728

[B26] Gimenez E. Salinas M. Manzano Agugliaro F. (2018). Worldwide research on plant defense against biotic stresses as improvement for sustainable agriculture. Sustainability 10, 391. doi: 10.3390/su10020391

[B27] Gleason C. Foley R. C. Singh K. B. (2011). Mutant analysis in arabidopsis provides insight into the molecular mode of action of the auxinic herbicide dicamba. PloS One 6, e17245. doi: 10.1371/journal.pone.0017245 21408147 PMC3050828

[B28] Gomes G. L. B. Scortecci K. C. (2021). Auxin and its role in plant development: Structure, signalling, regulation and response mechanisms. Plant Biol. 23, 894–904. doi: 10.1111/plb.13303 34396657

[B29] Gorina S. Ogorodnikova A. Mukhtarova L. Toporkova Y. (2022). Gene expression analysis of potato (*Solanum tuberosum* l.) lipoxygenase cascade and oxylipin signature under abiotic stress. Plants-Basel 11, 683. doi: 10.3390/plants11050683 35270153 PMC8912661

[B30] Gray W. M. Muskett P. R. Chuang H. W. Parker J. E. (2003). Arabidopsis SGT1b is required for SCF^TIR1^-mediated auxin response. Plant Cell 15, 1310–1319. doi: 10.1105/tpc.010884 12782725 PMC156368

[B31] Grossmann K. Scheltrup F. Kwiatkowski J. Caspar G. (1996). Induction of abscisic acid is a common effect of auxin herbicides in susceptible plants. J. Plant Physiol. 149, 475–478. doi: 10.1016/S0176-1617(96)80153-2

[B32] Guo F. Huang Y. Qi P. Lian G. Hu X. Han N. . (2021). Functional analysis of auxin receptor OsTIR1/OsAFB family members in rice grain yield, tillering, plant height, root system, germination, and auxinic herbicide resistance. New Phytol. 229, 2676–2692. doi: 10.1111/nph.17061 33135782

[B33] He X. J. Mu R. L. Cao W. H. Zhang Z. G. Zhang J. S. Chen S. Y. (2005). AtNAC2, a transcription factor downstream of ethylene and auxin signaling pathways, is involved in salt stress response and lateral root development. Plant J. 44, 903–916. doi: 10.1111/j.1365-313X.2005.02575.x 16359384

[B34] He Q. Yang L. Hu W. Zhang J. Xing Y. (2018). Overexpression of an auxin receptor OsAFB6 significantly enhanced grain yield by increasing cytokinin and decreasing auxin concentrations in rice panicle. Sci. Rep. 8, 14051. doi: 10.1038/s41598-018-32450-x 30232356 PMC6145926

[B35] Iglesias M. J. Terrile M. C. Bartoli C. G. D'Ippolito S. Casalongue C. A. (2010). Auxin signaling participates in the adaptative response against oxidative stress and salinity by interacting with redox metabolism in arabidopsis. Plant Mol. Biol. 74, 215–222. doi: 10.1007/s11103-010-9667-7 20661628

[B36] Iglesias M. J. Terrile M. C. Windels D. Lombardo M. C. Bartoli C. G. Vazquez F. . (2014). MiR393 regulation of auxin signaling and redox-related components during acclimation to salinity in arabidopsis. PloS One 9, e107678. doi: 10.1371/journal.pone.0107678 25222737 PMC4164656

[B37] Jagdale S. S. Joshi R. S. (2019). Facilitator roles of viruses in enhanced insect resistance to biotic stress. Curr. Opin. Insect Sci. 33, 111–116. doi: 10.1016/j.cois.2019.05.008 31358189

[B38] Jain M. Nijhawan A. Arora R. Agarwal P. Ray S. Sharma P. . (2007). F-box proteins in rice. genome-wide analysis, classification, temporal and spatial gene expression during panicle and seed development, and regulation by light and abiotic stress. Plant Physiol. 143, 1467–1483. doi: 10.1104/pp.106.091900 17293439 PMC1851844

[B39] Jin L. Qin Q. Q. Wang Y. Pu Y. Y. Liu L. F. Wen X. . (2016). Rice dwarf virus P2 protein hijacks auxin signaling by directly targeting the rice OsIAA10 protein, enhancing viral infection and disease development. PloS Pathog. 12, e1005847. doi: 10.1371/journal.ppat.1005847 27606959 PMC5015840

[B40] Khan M. R. Khan M. W. (1995). Effects of ammonia and root-knot nematode on tomato. Agr. Ecosyst. Environ. 53, 71–81. doi: 10.1016/0167-8809(94)00553-Q

[B41] Koide R. T. Dickie I. A. Goff M. D. (1999). Phosphorus deficiency, plant growth and the phosphorus efficiency index. Funct. Ecol. 13, 733–736. doi: 10.1046/j.1365-2435.1999.00363.x

[B42] Liu Y. J. Li D. Zhang S. Q. Zhang L. P. Gong J. Li Y. H. . (2022). Integrated analysis of microarray, small RNA, and degradome datasets uncovers the role of MicroRNAs in temperature-sensitive genic male sterility in wheat. Int. J. Mol. Sci. 23, 8057. doi: 10.3390/ijms23158057 PMC9332412 35897633

[B43] Liu J. N. Ma X. M. Yan L. P. Liang Q. Fang H. C. Wang C. X. . (2022). MicroRNA and degradome profiling uncover defense response of *fraxinus velutina* torr. to salt stress. Front. Plant Sci. 13, 847853. doi: 10.3389/fpls.2022.847853 35432418 PMC9011107

[B44] Liu H. B. Zhu R. Shu K. Lv W. X. Wang S. Wang C. L. (2022). Aluminum stress signaling, response, and adaptive mechanisms in plants. Plant Signal. Behav. 17, 2057060. doi: 10.1080/15592324.2022.2057060 35467484 PMC9045826

[B45] Llorente F. Muskett ,Sanchez-Vallet P. ,Lopez A. ,Ramos G. ,Sanchez-Rodriguez B. . (2008). Repression of the auxin response pathway increases arabidopsis susceptibility to necrotrophic fungi. Mol. Plant 1, 496–509. doi: 10.1093/mp/ssn025 19825556

[B46] Lo Gullo M. A. Salleo S. (1988). Different strategies of drought resistance in three mediterranean sclerophyllous trees growing in the same environmental conditions. New Phytol. 108, 267–276. doi: 10.1111/j.1469-8137.1988.tb04162.x 33873932

[B47] Luo M. Gao J. Peng H. Pan G. T. Zhang Z. M. (2014). MiR393-targeted TIR1-like (F-box) gene in response to inoculation to R.Solani in zea mays. Acta Physiol. Plant 36, 1283–1291. doi: 10.1007/s11738-014-1509-9

[B48] Lu Y. B. Qi Y. P. Yang L. T. Guo P. Li Y. Chen L. S. (2015). Boron-deficiency-responsive micrornas and their targets in citrus sinensis leaves. BMC Plant Biol. 15, 271. doi: 10.1186/s12870-015-0642-y 26538180 PMC4634795

[B49] Martin E. C. Sukarta O. C. A. Spiridon L. Grigore L. G. Constantinescu V. Tacutu R. . (2020). LRRpredictor-a new LRR motif detection method for irregular motifs of plant NLR proteins using an ensemble of classifiers. Genes 11, 286. doi: 10.3390/genes11030286 32182725 PMC7140858

[B50] Mayzlish-Gat i. De-Cuyper C. Goormachtig S. Beeckman T. Vuylsteke M. Brewer P. B. . (2012). Strigolactones are involved in root response to low phosphate conditions in arabidopsis. Plant Physiol. 160, 1329–1341. doi: 10.1104/pp.112.202358 22968830 PMC3490576

[B51] McClerklin S. A. Lee S. G. Harper C. P. Nwumeh R. Jez J. M. Kunkel. B. N. (2018). Indole-3-acetaldehyde dehydrogenase-dependent auxin synthesis contributes to virulence of pseudomonas syringae strain DC3000. PloS Pathog. 14, e1006811. doi: 10.1371/journal.ppat.1006811 29293681 PMC5766252

[B52] Mendoza-Soto A. B. Sanchez F. Hernandez G. (2012). MicroRNAs as regulators in plant metal toxicity response. Front. Plant Sci. 3, 105. doi: 10.3389/fpls.2012.00105 22661980 PMC3356851

[B53] Meng N. Tan X. Caldeon-Villalobos L. I. A. Estelle M. (2008). Mechanism of auxin perception by the SCF-TIR1 ubiquitin ligase. FASEB J. 22, 640–645. 10.1038/nature05731 17410169

[B54] Moradi A. El-Shetehy M. Gamir J. Austerlitz T. Dahlin P. Wieczorek K. . (2021). Expression of a fungal lectin in arabidopsis enhances plant growth and resistance toward microbial pathogens and a plant-parasitic nematode. Front. Plant Sci. 12, 657451. doi: 10.3389/fpls.2021.657451 33897746 PMC8063123

[B55] Muchate N. S. Nikalje G. C. Rajurkar N. S. Suprasanna P. Nikam T. D. (2016). Plant salt stress: Adaptive responses, tolerance mechanism and bioengineering for salt tolerance. Bot. Rev. 82, 371–406. doi: 10.1007/s12229-016-9173-y

[B56] Munoz A. Mangano S. Toribio R. Fernandez-Calvino L. del Pozo J. C. Mar Castellano M. (2022). The co-chaperone hop participates in TIR1 stabilisation and in auxin response in plants. Plant Cell Environ. 45, 2508–2519. doi: 10.1111/pce.14366 35610185 PMC9541403

[B57] Navarro L. Dunoyer P. Jay F. Arnold B. Dharmasiri N. Estelle M. . (2006). A plant miRNA contributes to antibacterial resistance by repressing auxin signaling. Science 312, 436–439. doi: 10.1126/science.1126088 16627744

[B58] Ortiz-Castro R. Diaz-Perez C. Martinez-Trujillo M. del Rio R. E. Campos-Garcia J. Lopez-Bucio J. (2011). Transkingdom signaling based on bacterial cyclodipeptides with auxin activity in plants. PNAS 108, 7253–7258. doi: 10.1073/pnas.1006740108 21482761 PMC3084137

[B59] Ozga J. A. Jayasinghege C. P. A. Kaur H. Gao L. C. Nadeau C. D Reinecke D. M. . (2022). Auxin receptors as integrators of developmental and hormonal signals during reproductive development in pea. J. Exp. Bot. 73, 4094–4112. doi: 10.1093/jxb/erac152 35395070

[B60] Padmanabhan M. S. Gorepoker S. P. Golem S. Shiferaw H. and Culver J. N. (2005). Interaction of the tobacco mosaic virus replicase protein with the Aux/IAA protein PAPI/IAA26 is associated with disease development. J. Virol. 79, 2549–2558. doi: 10.1128/JVI.79.4.2549-2558.2005 15681455 PMC546588

[B61] Pan J. Fujioka S. Peng J. Chen J. Li G. Chen R. (2009). The E3 ubiquitin ligase SCF^TIR1/AFB^ and membrane sterols play key roles in auxin regulation of endocytosis, recycling, and plasma membrane accumulation of the auxin efflux transporter PIN2 in arabidopsis thaliana. Plant Cell 21, 568–580. doi: 10.1105/tpc.108.061465 19218398 PMC2660622

[B62] Park M. Li Q. Shcheynikov N. Muallem S. Zeng W. Z. (2005). Borate transport and cell growth and proliferation - not only in plants. Cell Cycle 4, 24–26. doi: 10.4161/cc.4.1.1394 15611652

[B63] Parry G. Calderon-Villalobos L. I. Prigge M. Peret B. Dharmasiri S. Itoh H. . (2009). Complex regulation of the TIR1/AFB family of auxin receptors. PNAS 106, 22540–22545. doi: 10.1073/pnas.0911967106 20018756 PMC2799741

[B64] Perez-Torres C. ,Lopez-Bucio A. ,Cruz-Ramirez J. ,Ibarra-Laclette A. ,Dharmasiri E. ,Estelle S. . (2008). Phosphate availability alters lateral root development in arabidopsis by modulating auxin sensitivity *via* a mechanism involving the TIR1 auxin receptor. Plant Cell 20, 3258–3272. doi: 10.1105/tpc.108.058719 19106375 PMC2630440

[B65] Perez Torres C. A. Lopez Bucio J. Herrera Estrella L. (2009). Low phosphate signaling induces changes in cell cycle gene expression by increasing auxin sensitivity in the arabidopsis root system. Plant Signal. Behav. 4, 781–783. doi: 10.4161/psb.4.8.9230 19820337 PMC2801399

[B66] Prigge M. J. Greenham K. Zhang Y. Santner A. Castillejo C. Mutka A. M. . (2016). The arabidopsis auxin receptor f-box proteins AFB4 and AFB5 are required for response to the synthetic auxin picloram. Genes Genom. Genet. 6, 1383–1390. doi: 10.1534/g3.115.025585 PMC4856089 26976444

[B67] Prigge M. J. Platre M. Kadakia N. Zhang Y. Greenham K. Szutu W. . (2020). Genetic analysis of the arabidopsis TIR1/AFB auxin receptors reveals both overlapping and specialized functions. Elife 9, e54740. doi: 10.7554/eLife.54740.sa2 32067636 PMC7048394

[B68] Quint M. Gray W. M. (2006). Auxin signaling. Curr. Opin. Plant Biol. 9, 448–453. doi: 10.1016/j.pbi.2006.07.006 16877027 PMC2424235

[B69] Rast-Somssich M. I. Zadnikova P. Schmid S. Kieffer M. Kepinski S. Simon R. (2017). The arabidopsis JAGGED LATERAL ORGANS (JLO) gene sensitizes plants to auxin. J. Exp. Bot. 68, 2741–2755. doi: 10.1093/jxb/erx131 28472464 PMC5853575

[B70] Rejeb I. B. Pastor V. Mauch Mani B. (2014). Plant responses to simultaneous biotic and abiotic stress: Molecular mechanisms. Plants-Basel 3, 458–475. doi: 10.3390/plants3040458 27135514 PMC4844285

[B71] Rogato A. Valkov V. T. Nadzieja M. Stougaard J. Chiurazzi M. (2021). The lotus japonicus AFB6 gene is involved in the auxin dependent root developmental program. Int. J. Mol. Sci. 22, 8495. doi: 10.3390/ijms22168495 34445201 PMC8395167

[B72] Ruegger M. Dewey E. Gray W. M. Hobbie L. Turner J. Estelle M. (1998). The TIR1 protein of arabidopsis functions in auxin response and is related to human SKP2 and yeast Grr1p. Gene Dev. 12, 198–207. doi: 10.1101/gad.12.2.198 9436980 PMC316440

[B73] Ruiz-Herrera L. F. Lopez-Bucio J. (2013). Aluminum induces low phosphate adaptive responses and modulates primary and lateral root growth by differentially affecting auxin signaling in arabidopsis seedlings. Plant Soil 371, 593–609. doi: 10.1007/s11104-013-1722-0

[B74] Sakamoto T. Kimura S. (2018). Plant temperature sensors. Sensors 18, 4365. doi: 10.3390/s18124365 30544707 PMC6308845

[B75] Salehin M. Bagchi R. Estelle M. (2015). SCF^TIR1/AFB^-based auxin perception: Mechanism and role in plant growth and development. Plant Cell 27, 9–19. doi: 10.1105/tpc.114.133744 25604443 PMC4330579

[B76] Sattar S. Addo-Quaye C. Thompson G. A. (2016). miRNA-mediated auxin signalling repression during vat-mediated aphid resistance in cucumis melo. Plant Cell Environ. 39, 1216–1227. doi: 10.1111/pce.12645 26437210

[B77] Seah S. Telleen A. C. Williamson V. M. (2007). Introgressed and endogenous mi-1 gene clusters in tomato differ by complex rearrangements in flanking sequences and show sequence exchange and diversifying selection among homologues. Theor. Appl. Genet. 114, 1289–1302. doi: 10.1007/s00122-007-0519-z 17318492

[B78] Seifi A. Visser R. G. F. Bai Y. (2011). Differential expression of TIR-like genes embedded in the mi-1 gene cluster in nematode-resistant and -susceptible tomato roots. J. Plant Pathol. 93, 701–706.

[B79] Sharma L. Dalal M. Verma R. K. Kumar S. V. V. Yadav S. K. Pushkar S. . (2018). Auxin protects spikelet fertility and grain yield under drought and heat stresses in rice. Environ. Exp. Bot. 150, 9–24. doi: 10.1016/j.envexpbot.2018.02.013

[B80] Sheedy C. Yau K. Y. F. Hirama T. MacKenzie C. R. Hall J. C. (2006). Selection, characterization, and CDR shuffling of naive llama single-domain antibodies selected against auxin and their cross-reactivity with auxinic herbicides from four chemical families. J. Agr. Food Chem. 54, 3668–3678. doi: 10.1021/jf060219i 19127743

[B81] Shimizu-Mitao Y. Kakimoto T. (2014). Auxin sensitivities of all arabidopsis Aux/IAAs for degradation in the presence of every TIR1/AFB. Plant Cell Physiol. 55, 1450–1459. doi: 10.1093/pcp/pcu077 24880779

[B82] Shi G. G. Wang S. S. Wang P. Zhan J. J. Tang Y. Zhao G. . (2022). Cotton miR393-TIR1 module regulates plant defense against verticillium dahliae via auxin perception and signaling. Front. Plant Sci. 13, 888703. doi: 10.3389/fpls.2022.888703 35592575 PMC9111529

[B83] Shi H. Ye T. Han N. Bian H. Liu X. Chan Z. (2015). Hydrogen sulfide regulates abiotic stress tolerance and biotic stress resistance in arabidopsis. J. Integr. Plant Biol. 57, 628–640. doi: 10.1111/jipb.12302 25329496

[B84] Shu W. Liu Y. Guo Y. Zhou ,Zhang H. ,Zhao J. . (2015). A populus TIR1 gene family survey reveals differential expression patterns and responses to 1-naphthaleneacetic acid and stress treatments. Front. Plant Sci. 6, 719. doi: 10.3389/fpls.2015.00719 26442033 PMC4585115

[B85] Singh S. Kumar V. Kapoor D. Kumar S. Singh S. Dhanjal D. S. . (2020). Revealing on hydrogen sulfide and nitric oxide signals co-ordination for plant growth under stress conditions. Physiol. Plantarum 168, 301–317. doi: 10.1111/ppl.13066 31264712

[B86] Strader L. C. Zhao Y. (2016). Auxin perception and downstream events. Curr. Opin. Plant Biol. 33, 8–14. doi: 10.1016/j.pbi.2016.04.004 27131035 PMC5050066

[B87] Striegel S. Oliveira M. C. Arneson N. Conley S. P. Stoltenberg D. E. Werle R. (2021). Spray solution pH and soybean injury as influenced by synthetic auxin formulation and spray additives. Weed Technol. 35, 113–127. doi: 10.1017/wet.2020.89

[B88] Su Y. X. Wang G. L. Huang Z. Y. Hu L. L. Fu T. Wang X. Y. . (2022). Silencing GhIAA43, a member of cotton AUX/IAA genes, enhances wilt resistance *via* activation of salicylic acid-mediated defenses. Plant Sci. 314, 111126. doi: 10.1016/j.plantsci.2021.111126 34895552

[B89] Su P. Zhao L. Li W. Zhao J. Yan J. Ma X. . (2021). Integrated metabolo-transcriptomics and functional characterization reveals that the wheat auxin receptor TIR1 negatively regulates defense againstfusarium graminearum. J. Integr. Plant Biol. 63, 340–352. doi: 10.1111/jipb.12992 32678930

[B90] Takato S. Kakei Y. Mitsui M. Ishida Y. Suzuki M. Yamazaki C. . (2017). Auxin signaling through SCF^TIR1/AFBs^ mediates feedback regulation of IAA biosynthesis. Biosci. Biotech. Bioch. 81, 1320–1326. doi: 10.1080/09168451.2017.1313694 28406060

[B91] Termorshuizen A. J. (2016). Ecology of fungal plant pathogens. Microbiol. Spectr. 4. doi: 10.1128/microbiolspec.FUNK-0013-2016 28087933

[B92] Todd O. E. Figueiredo M. R. A. Morran S. Soni N. Preston C. Kubes M. F. . (2020). Synthetic auxin herbicides: Finding the lock and key to weed resistance. Plant Sci. 300, 110631. doi: 10.1016/j.plantsci.2020.110631 33180710

[B93] Verma V. Ravindran P. Kumar P. P. (2016). Plant hormone-mediated regulation of stress responses. BMC Plant Biol. 16, 86. doi: 10.1186/s12870-016-0771-y 27079791 PMC4831116

[B94] Vidal E. A. Araus V. Lu C. Parry G. Green P. J. Coruzzi G. M. . (2010). Nitrate-responsive miR393/AFB3 regulatory module controls root system architecture in arabidopsis thaliana. PNAS 107, 4477–4482. doi: 10.1073/pnas.0909571107 20142497 PMC2840086

[B95] Vidal E. A. Moyano T. C. Riveras E. Contreras-Lopez O. Gutierrez R. A. (2013). Systems approaches map regulatory networks downstream of the auxin receptor AFB3 in the nitrate response of arabidopsis thaliana roots. PNAS 110, 12840–12845. doi: 10.1073/pnas.1310937110 23847199 PMC3732920

[B96] Villalobos L. Lee S. De Oliveira C. Ivetac A. Brandt W. Armitage L. . (2012). A combinatorial TIR1/AFB-Aux/IAA co-receptor system for differential sensing of auxin. Nat. Chem. Biol. 8, 477–485. doi: 10.1038/nchembio.926 22466420 PMC3331960

[B97] Vitor S. C. Duarte G. T. Saviani E. E. Vincentz M. G. A. Oliveira H. C. Salgado I. (2013). Nitrate reductase is required for the transcriptional modulation and bactericidal activity of nitric oxide during the defense response of arabidopsis thaliana against pseudomonas syringae. Planta 238, 475–486. doi: 10.1007/s00425-013-1906-0 23748675

[B98] Vo K. T. X. Rahman M. M. Rahman M. M. Trinh K. T. T. Kim S. T. Jeon J. S. (2021). Proteomics and metabolomics studies on the biotic stress responses of rice: An update. Rice 14, 30. doi: 10.1186/s12284-021-00461-4 33721115 PMC7960847

[B99] Walsh T. A. Neal R. Merlo A. O. Honma M. Hicks G. R. Wolff K. . (2006). Mutations in an auxin receptor homolog AFB5 and in SGT1b confer resistance to synthetic picolinate auxins and not to 2,4-dichlorophenoxyacetic acid or indole-3-acetic acid in arabidopsis. Plant Physiol. 142, 542–552. doi: 10.1104/pp.106.085969 16920877 PMC1586033

[B100] Wang F. Niu H. Xin D. Long Y. Wang G. Liu Z. . (2021). OsIAA18, an Aux/IAA transcription factor gene, is involved in salt and drought tolerance in rice. Front. Plant Sci. 12, 738660. doi: 10.3389/fpls.2021.738660 34868122 PMC8637529

[B101] Wang D. Pajerowska-Mukhtar K. Culler A. H. Dong X. N. (2007). Salicylic acid inhibits pathogen growth in plants through repression of the auxin signaling pathway. Curr. Biol. 17, 1784–1790. doi: 10.1016/j.cub.2007.09.025 17919906

[B102] Wang P. H. Wu P. C. Huang R. C. Chung K. R. (2020). The role of a nascent polypeptide-associated complex subunit alpha in siderophore biosynthesis, oxidative stress response, and virulence in *Alternaria alternata* . Mol. Plant Microbe In. 33, 668–679. doi: 10.1094/MPMI-11-19-0315-R 31928525

[B103] Wang R. Zhang Y. Kieffer M. Yu H. Kepinski S. Estelle M. (2016). HSP90 regulates temperature-dependent seedling growth in arabidopsis by stabilizing the auxin co-receptor f-box protein TIR1. Nat. Commun. 7, 1026. doi: 10.1038/ncomms10269 PMC4728404 26728313

[B104] Wang S. K. Zhang S. N. Sun C. D. Xu Y. X. Chen Y. Yu C. L. . (2014). Auxin response factor (OsARF12), a novel regulator for phosphate homeostasis in rice (*oryza sativa*). New Phytol. 201, 91–103. doi: 10.1111/nph.12499 24111723

[B105] Watanabe E. Mano S. Nomoto M. Tada Y. Hara-Nishimura I. Nishimura M. . (2016). HSP90 stabilizes auxin-responsive phenotypes by masking a mutation in the auxin receptor TIR1. Plant Cell Physiol. 57, 2245–2254. doi: 10.1093/pcp/pcw170 27816945

[B106] Wildermuth M. C. Dewdney J. Wu G. Ausubel F. M. (2001). Isochorismate synthase is required to synthesize salicylic acid for plant defence. Nature 414, 562–565. doi: 10.1038/35107108 11734859

[B107] Xia K. Wang R. Ou X. Fang Z. Tian C. Duan J. . (2012). OsTIR1 and OsAFB2 downregulation *via* OsmiR393 overexpression leads to more tillers, early flowering and less tolerance to salt and drought in rice. PloS One 7, 364–373. doi: 10.1371/journal.pone.0030039 PMC3254625 22253868

[B108] Xie Q. Frugis G. Colgan D. Chua N. H. (2000). Arabidopsis NAC1 transduces auxin signal downstream of TIR1 to promote lateral root development. Gene Dev. 14, 3024–3036. doi: 10.1101/gad.852200 11114891 PMC317103

[B109] Xu J. Q. Liu X. D. Napier R. Dong L. Y. Li J. (2022). Mode of action of a novel synthetic auxin herbicide halauxifen-methyl. Agronomy-Basel 12, 1659. doi: 10.3390/agronomy12071659

[B110] Yang C. W. Deng W. Tang N. Wang X. M. Yan F. Lin D. B. . (2013). Overexpression of ZmAFB2, the maize homologue of AFB2 gene, enhances salt tolerance in transgenic tobacco. Plant Cell Tiss. Org. 112, 171–179. doi: 10.1007/s11240-012-0219-5

[B111] Yang Y. Guo Y. (2018). Unraveling salt stress signaling in plants. J. Integr. Plant Biol. 60, 796–804. doi: 10.1111/jipb.12689 29905393

[B112] Yu H. Y. Huang S. T. Chen P. P. Ji M. J. Cui H. L. Chen J. C. . (2021). Different leaf-mediated deposition, absorbed and metabolism behaviors of 2,4-d isooctyl ester between Triticum aestivum and *Aegilops tauschii* coss. Pestic. Biochem. Phys. 175, 104848. doi: 10.1016/j.pestbp.2021.104848 33993966

[B113] Yu Z. Zhang F. Friml J. Ding Z. (2022). Auxin signaling: Research advances over the past 30 years. J. Integr. Plant Biol. 64, 371–392. doi: 10.1111/jipb.13225 35018726

[B114] Zhang R. M. Sun Y. K. Liu Z. Y. Jin W. Sun Y. (2017). Effects of melatonin on seedling growth, mineral nutrition, and nitrogen metabolism in cucumber under nitrate stress. J. Pineal. Res. 62, e12403. doi: 10.1111/jpi.12403 28226188

[B115] Zhang H. H. Tan X. X. Li L. L. He Y. Q. Hong G. J. Li J. M. . (2019). Suppression of auxin signalling promotes rice susceptibility to rice black streaked dwarf virus infection. Mol. Plant Pathol. 20, 1093–1104. doi: 10.1111/mpp.12814 31250531 PMC6640184

[B116] Zhang A. Y. Yang X. Lu J. Song F. Y. Sun J. H. Wang C. . (2021). OsIAA20, an Aux/IAA protein, mediates abiotic stress tolerance in rice through an ABA pathway. Plant Sci. 308, 110903. doi: 10.1016/j.plantsci.2021.110903 34034863

[B117] Zhao J. Yuan S. Zhou M. Yuan N. Li Z. Hu Q. . (2019). Transgenic creeping bentgrass overexpressing osa-mir393a exhibits altered plant development and improved multiple stress tolerance. Plant Biotechnol. J. 17, 233–251. doi: 10.1111/pbi.12960 29873883 PMC6330543

[B118] Zhu J. K. (2002). Salt and drought stress signal transduction in plants. Annu. Rev. Plant Biol. 53, 247–273. doi: 10.1146/annurev.arplant.53.091401.143329 12221975 PMC3128348

[B119] Zimaro T. Gottig N. Garavaglia B. S. Gehring C. Ottado J. (2011). Unraveling plant responses to bacterial pathogens through proteomics. J. Biomed. Biotechnol. 2011, 354801. doi: 10.1155/2011/354801 22131803 PMC3216475

